# Harnessing CRISPR/Cas Systems for DNA and RNA Detection: Principles, Techniques, and Challenges

**DOI:** 10.3390/bios14100460

**Published:** 2024-09-26

**Authors:** Heyjin Son

**Affiliations:** Korea Research Institute of Bioscience and Biotechnology, Daejeon 34141, Republic of Korea; heyjin@kribb.re.kr

**Keywords:** CRISPR/Cas, nucleic acids detection, diagnosis, biosensing

## Abstract

The emergence of CRISPR/Cas systems has revolutionized the field of molecular diagnostics with their high specificity and sensitivity. This review provides a comprehensive overview of the principles and recent advancements in harnessing CRISPR/Cas systems for detecting DNA and RNA. Beginning with an exploration of the molecular mechanisms of key Cas proteins underpinning CRISPR/Cas systems, the review navigates the detection of both pathogenic and non-pathogenic nucleic acids, emphasizing the pivotal role of CRISPR in identifying diverse genetic materials. The discussion extends to the integration of CRISPR/Cas systems with various signal-readout techniques, including fluorescence, electrochemical, and colorimetric, as well as imaging and biosensing methods, highlighting their advantages and limitations in practical applications. Furthermore, a critical analysis of challenges in the field, such as target amplification, multiplexing, and quantitative detection, underscores areas requiring further refinement. Finally, the review concludes with insights into the future directions of CRISPR-based nucleic acid detection, emphasizing the potential of these systems to continue driving innovation in diagnostics, with broad implications for research, clinical practice, and biotechnology.

## 1. Introduction

CRISPR/Cas systems have significantly advanced the field of molecular diagnostics, introducing unprecedent specificity and sensitivity for nucleic acid detection [1,2,3,4,5,6,7]. Initially discovered as the bacterial and archaeal adaptive immune systems, these systems target and cleave foreign nucleic acids, offering a robust framework for a range of applications beyond genome editing [8,9,10]. This review thoroughly examines the fundamental principles and latest developments in the application of CRISPR/Cas systems for DNA and RNA detection.

Starting with the molecular mechanisms that enable CRISPR/Cas systems to perform nucleic acid detection, this review focuses on various CRISPR-associated (Cas) effectors like Cas9, Cas12, Cas13, and Cas14. Each of these proteins possesses unique characteristics that make them suitable for specific detection tasks. CRISPR/Cas systems operate through guide RNAs (gRNAs) that direct Cas proteins to specific nucleic acid sequences, enabling cleavage and detection. By integrating CRISPR/Cas with amplification methods such as polymerase chain reaction (PCR) and isothermal amplification techniques, the sensitivity of detection assays has been significantly improved [11]. These combined techniques amplify target nucleic acids, facilitating their accurate detection via CRISPR-based systems, and allowing the identification of even trace amounts of nucleic acids.

The review explores various signal readout methods that complement CRISPR-based nucleic acid detection, each offering distinct advantages and utilities. For pathogenic nucleic acids, fluorescence-based readouts are known for their high sensitivity and are commonly used in laboratory environments. Electrochemical readouts provide portability and are easily incorporated into point-of-care (POC) diagnostic devices, while colorimetric readouts offer visual, qualitative results, making them ideal for rapid, on-site diagnostics [2]. For non-pathogenic nucleic acids, optical imaging systems, such as those incorporating electrochemical or surface plasmon resonance technologies, offer versatile and sensitive approaches for analyzing genetic materials in various contexts [12].

Despite these advancements, several challenges remain in the practical use of CRISPR-based nucleic acid detection. Amplification of target nucleic acids for a low limit of detection is essential but can introduce complexity and potential errors. Multiplexed detection, which involves the simultaneous identification of multiple targets, presents challenges in maintaining specificity and avoiding cross-reactivity. Quantitative detection, necessary for applications requiring precise measurement of nucleic acid quantities, also needs further refinement. Overcoming these challenges involves optimizing and refining CRISPR/Cas systems to expand their utility in diagnostics and beyond. Efforts to streamline amplification processes, enhance multiplexing capabilities, and improve quantitative accuracy are essential for the future development of CRISPR-based assays.

In summary, CRISPR/Cas systems have not only transformed genome editing but have also paved the way for significant advancements in nucleic acid detection. Offering high specificity, sensitivity, and versatility, CRISPR-based assays are set to become essential tools in molecular diagnostics, advancing both fundamental research and clinical applications. This review aims to provide a detailed overview of the principles, advancements, challenges, and future prospects of CRISPR-based nucleic acid detection, highlighting its transformative potential in the rapidly evolving field of biotechnology.

## 2. Fundamentals of CRISPR-Based Nucleic Acid Detection

**Figure 1:**
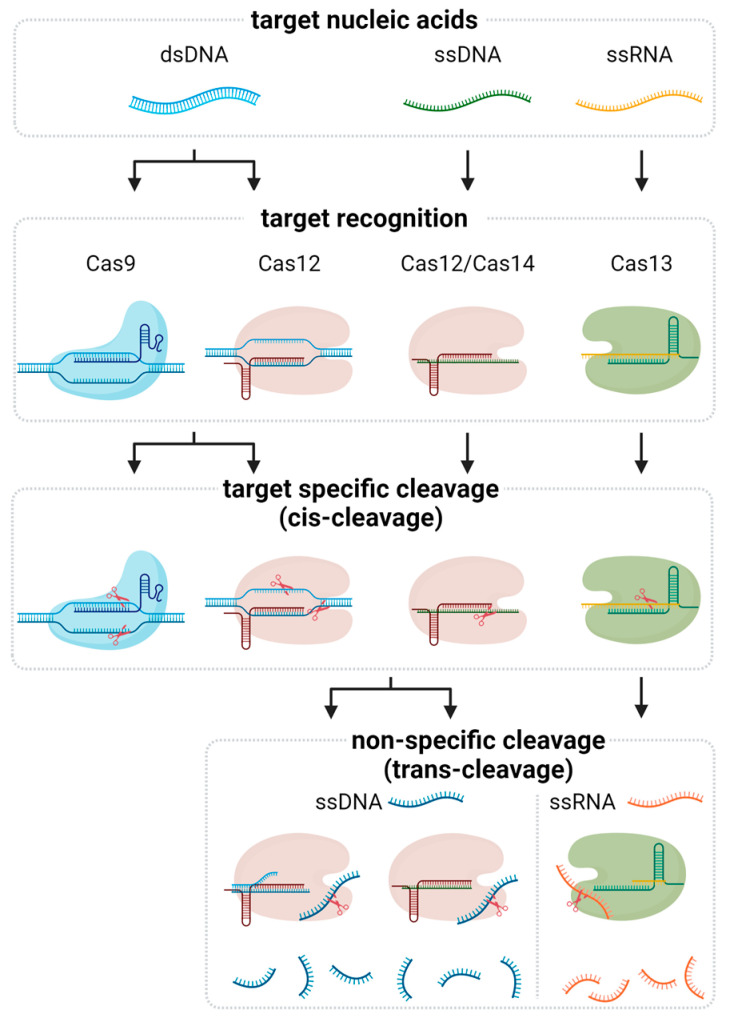
Molecular mechanism of CRISPR-associated nucleic acid detection. The Cas protein-gRNA complex first forms and binds to the target nucleic acid. Different Cas proteins bind depending on the type of target. The ribonucleoprotein (RNP) complex then cleaves the target, a process known as cis-cleavage. While the cleavage reaction mediated by Cas9 terminates at this stage, other Cas proteins, including Cas12, Cas13, and Cas14, further exhibit nonspecific cleavage of nearby nucleic acids, referred to as trans-cleavage. This trans-cleavage activity is a key mechanism for signal amplification in CRISPR-based diagnostics.

CRISPR-based nucleic acid detection leverages the targeting capabilities of Cas proteins to identify specific sequences within DNA or RNA. Among the various Cas effectors, Cas9, Cas12, Cas13, and Cas14 have been extensively utilized due to their unique properties and mechanisms of action (Figure 1 and Table 1).

### 2.1. Cas9

Cas9 is the most well-known CRISPR-associated protein, primarily recognized for its role in bacterial immunity. It operates through a single guide RNA (sgRNA), which is made up of CRISPR RNA (crRNA) and trans-activating crRNA (tracrRNA), directing Cas9 to specific DNA sequences adjacent to a protospacer adjacent motif (PAM), introducing a blunt-ended double-strand break (DSB) [13,14]. This property has been harnessed for nucleic acid detection by coupling Cas9 with signal amplification techniques. Cas9-mediated cleavage triggers detectable signals via electrochemical or fluorescence methods, making it powerful for detecting genetic variations and pathogen-specific sequences due to its high specificity and ability to discriminate single nucleotide polymorphisms (SNPs).

### 2.2. Cas12

Cas12 proteins, such as Cas12a, expand the versatility of CRISPR-based detection. Unlike Cas9, Cas12a requires a T-rich PAM sequence and generates staggered cuts with 5′ overhangs. One of the notable features of Cas12a is its trans-cleavage activity, where it nonspecifically cleaves single-stranded DNA (ssDNA) upon binding to its target DNA [15,16]. This collateral cleavage activity has been exploited in diagnostic assays like DETECTR, where the presence of target DNA activates Cas12a to cleave DNA reporters, producing a fluorescent or colorimetric signal. The simplicity and robustness of Cas12a-based detection systems facilitate their application in POC diagnostics.

### 2.3. Cas13

Cas13, particularly Cas13a, targets RNA rather than DNA, expanding the scope of CRISPR diagnostics to include RNA viruses and other RNA molecules. Cas13a, guided by a crRNA, binds to its target RNA and activates RNase activity, leading to the nonspecific cleavage of nearby RNA [17]. This collateral cleavage is central to the SHERLOCK platform, enabling sensitive detection of RNA sequences through the cleavage of labeled RNA reporters. The ability of Cas13a to detect RNA directly without the need for reverse transcription is invaluable for rapid and accurate detection of RNA viruses like SARS-CoV-2.

### 2.4. Cas14

Cas14 proteins are characterized by their exceptionally small size and ability to target ssDNA without requiring a PAM sequence [18]. Cas14’s high specificity and sensitivity make it suitable for detecting small and fragmented DNA samples. Similar to Cas12, Cas14 exhibits trans-cleavage activity, generating detectable signals in the presence of target ssDNA. The small size of Cas14 also facilitates incorporation into various diagnostic platforms, potentially enhancing the portability and scalability of CRISPR-based detection technologies.

In summary, the distinct properties of Cas9, Cas12, Cas13, and Cas14 proteins provide a diverse toolkit for CRISPR-based nucleic acid detection. Each protein’s unique mechanism and specificity allow for tailored diagnostic assays suited for different types of nucleic acids and detection requirements. By leveraging these capabilities, CRISPR-based detection systems offer rapid, sensitive, and specific diagnostic solutions, paving the way for advancements in medical diagnostics, pathogen surveillance, and beyond.

## 3. CRISPR-Based Detection for Nucleic Acids

CRISPR-based detection systems offer robust and versatile methods for identifying nucleic acids by utilizing their unique molecular mechanism. This section is divided into two categories: the detection of DNA and RNA, addressing pathogen and non-pathogen detection. These subsections explore the unique applications of CRISPR/Cas systems for detecting nucleic acids in various diagnostic contexts, highlighting the innovative approaches used to achieve high specificity, sensitivity, and rapidity in molecular diagnostics. A brief description of amplification methods used in CRISPR-based nucleic acid detection are listed in Table 2.

### 3.1. Detection of DNA

The detection of DNA using CRISPR-based systems has significantly advanced various applications, including pathogen identification and genetic analysis. This subsection deals with the detection of pathogenic DNA and non-pathogenic DNA. Pathogenic DNA detection focuses on identifying harmful genetic material from viruses, bacteria, and other microorganisms, enabling rapid diagnosis and disease monitoring. Non-pathogenic DNA detection, on the other hand, addresses the identification of genetic markers, mutations, and other significant genetic variations that are essential for oncology, genetic engineering, and personalized medicine. These subsections illustrate the versatility and precision of CRISPR/Cas technologies in different diagnostic scenarios.

#### 3.1.1. Pathogenic DNA Detection

CRISPR-based methods have significantly enhanced the detection of pathogenic DNA due to their high specificity and sensitivity. Cas9 and Cas12 are pivotal in these applications. Cas9, guided by a sgRNA, can target and cleave specific pathogen sequences. This cleavage event can be detected through amplification and readout methods, facilitating the identification of pathogens like human papillomavirus (HPV) and various pathogenic bacteria [19,20,21,22,23]. The high specificity of Cas9 makes it ideal for distinguishing SNPs within pathogen genomes, which is crucial for identifying strains and resistance markers.

Cas12 and Cas14, with its trans-cleavage activity, further extends the capabilities of CRISPR-based diagnostics. The collateral activity has been harnessed in diagnostic platforms like DETECTR [18,24,25]. In DETECTR, the presence of pathogenic DNA triggers Cas12 and Cas14 to cleave a reporter DNA molecule, generating a fluorescent or colorimetric signal. This method has been successfully applied to detect pathogens such as African swine fever virus and HPV, offering rapid and sensitive diagnostics suitable for POC settings [26,27,28,29,30,31,32].

#### 3.1.2. Non-Pathogenic DNA Detection

The detection of non-pathogenic DNA using CRISPR/Cas systems focuses on genetic markers, mutations, and other relevant DNA sequences. High specificity of Cas proteins enables detecting SNPs within the genome successfully.

One significant application of Cas9 is in cancer diagnostics, where detecting circulating tumor DNA (ctDNA) is essential [33,34]. Techniques like CRISPR-mediated ultrasensitive detection (CUT-PCR) use Cas9 to enrich and amplify specific DNA fragments, enhancing the detection of low-abundance sequences. This method can identify genetic mutations and alterations in ctDNA, providing valuable information for cancer diagnosis and monitoring. Additionally, Cas9 has been applied to the detection of mitochondrial DNA (mtDNA) mutations using a proximity ligation assay (CasPLA), which enables the imaging of mtDNA mutations at single-molecule resolution through fluorescence detection. This approach is particularly useful for studying mitochondrial genetics and related diseases [35].

Cas12 also plays a crucial role in non-pathogenic DNA detection, particularly in identifying SNPs and DNA methylation patterns [36,37,38,39]. These features are vital for understanding genetic diversity and epigenetic modifications. By combining Cas12 with isothermal amplification methods, sensitive and simple assays were developed for an ideal use in low-resource settings. These advancements facilitate the study of genetic traits, agricultural biotechnology, and personalized medicine.

### 3.2. Detection of RNA

CRISPR-based RNA detection has opened new avenues for diagnosing and monitoring various diseases, especially those caused by RNA viruses. This section explores two main areas: the detection of pathogenic RNA and non-pathogenic RNA. Pathogenic RNA detection is crucial for identifying and managing viral infections such as SARS-CoV-2, Zika, and Influenza, providing tools for rapid and accurate disease surveillance. Non-pathogenic RNA detection focuses on identifying regulatory RNAs, such as microRNAs and messenger RNAs, which are vital for understanding gene expression patterns and cellular responses. These subsections highlight the innovative use of CRISPR/Cas systems in enhancing the sensitivity and specificity of RNA diagnostics.

#### 3.2.1. Pathogenic RNA Detection

Similar to pathogenic DNA detection, Cas9 and Cas12 are employed to detect RNA viruses such as SARS-CoV-2 and Zika by combining with a reverse transcription [40,41,42,43,44,45]. In contrast, Cas13 proteins are capable of directly identifying RNA, eliminating the reverse transcription process. The indiscriminate ssRNA cleavage upon binding of target RNA to Cas13 is central to the SHERLOCK platform, which is used to detect RNA viruses like Zika, Dengue, and SARS-CoV-2 [46,47,48,49]. SHERLOCK combines Cas13a with amplification techniques such as recombinase polymerase amplification (RPA) or PCR to increase the sensitivity of RNA detection. The presence of target RNA leads to Cas13a-mediated cleavage of a fluorescent reporter, producing a detectable signal indicative of the pathogen.

This method’s ability to directly detect RNA without reverse transcription simplifies the process and speeds up diagnosis. The high sensitivity and specificity of SHERLOCK make it a powerful tool for identifying emerging viral threats quickly and accurately.

#### 3.2.2. Non-Pathogenic RNA Detection

CRISPR-based techniques for non-pathogenic RNA detection focus on identifying regulatory RNAs and other RNA molecules important for gene expression and regulation [50,51,52,53,54,55]. Cas13a’s ability to cleave RNA has been employed to detect microRNAs (miRNAs), which play pivotal roles in regulating gene expression and are significant biomarkers for diseases such as cancer [56,57]. Using catalytic hairpin assembly (CHA) to amplify the signal, the Cas12a-based assays can detect miRNAs at very low concentrations, providing valuable diagnostic information [58].

Furthermore, CRISPR/Cas systems are utilized to detect messenger RNAs (mRNAs), which are indicative of gene expression levels [54,59,60]. By targeting specific mRNA sequences, researchers can monitor cellular responses and understand gene regulation mechanisms. This application is particularly useful in studying developmental biology, disease mechanisms, and therapeutic responses, offering insights into the dynamic processes governing cellular function.

## 4. Signal Readout Methods for CRISPR-Based Nucleic Acid Detection

**Figure 2:**
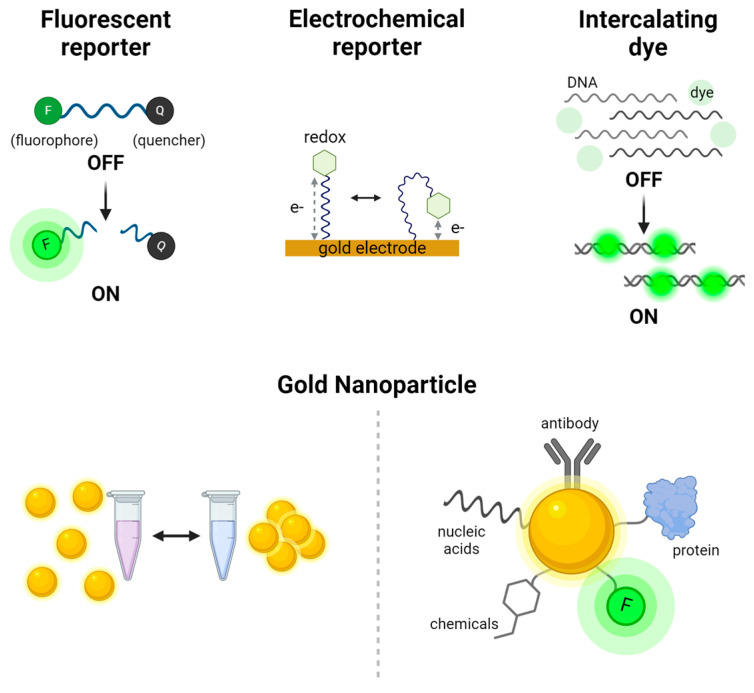
Signal production systems used in CRISPR-based nucleic acid detection. The fluorescent reporter system relies on energy transfer between a fluorophore (F) and a quencher (Q), where fluorescence is generated upon cleavage of the reporter. The electrochemical reporter system measures changes in electrical signals dependent on the distance between a redox molecule and gold electrode. Intercalating dyes, such as SYBR Green, bind to double-stranded DNA and emit fluorescence upon intercalation. Gold nanoparticles serve as versatile reporters; they can be visualized by color changes through aggregation visible to the naked eye and also be conjugated with a variety of molecules, including antibodies, proteins, nucleic acids, fluorophores, and chemicals, for diverse biosensing applications.

**Figure 3:**
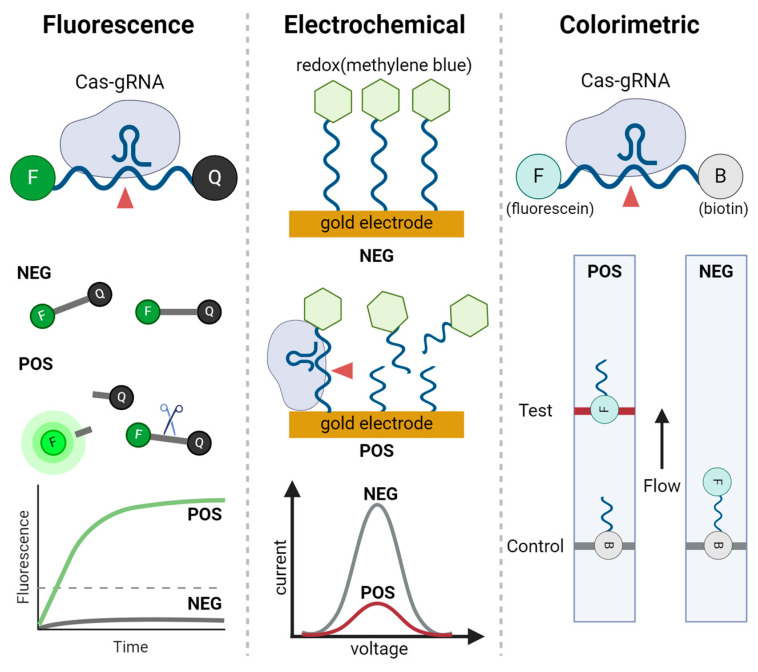
Signal readout methods used in CRISPR-based nucleic acid detection. Three major readout methods are illustrated: fluorescence, electrochemical, and colorimetric. In fluorescence readouts, the Cas-gRNA complex cleaves fluorescent reporters, producing a fluorescence signal. The graph shows the increase in fluorescence over time for positive sample (POS) compared to negative samples (NEG). In electrochemical readouts, redox molecules are conjugated to nucleic acids and immobilized on a gold electrode. When CRISPR complex interacts with the redox-labeled nucleic acids, a detectable change in current is produced, as shown in the voltage-current graph for positive and negative samples. The colorimetric readout, typically in the form of a lateral flow assay (LFA), reports positive results by capturing cleaved products labeled with fluorescein, producing visible bands on the test line, while biotin-labeled parts produce visible bands on the control line.

**Figure 4:**
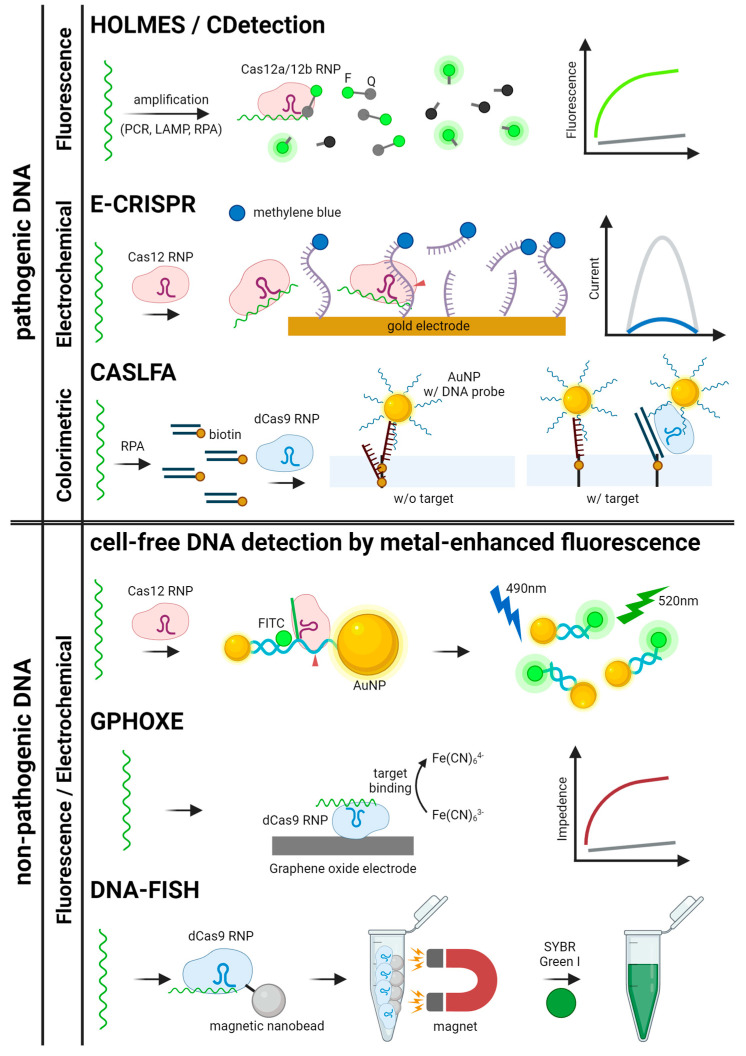
Schematics for CRISPR-based detection systems for pathogenic and non-pathogenic DNA. The detection systems are categorized by signal readout methods. HOLMES, CDetection, E-CRISPR, CASLFA, GPHOXE, DNA-FISH, and metal-enhanced fluorescence detection are illustrated [23,27,29,34,37,88,89].

**Figure 5:**
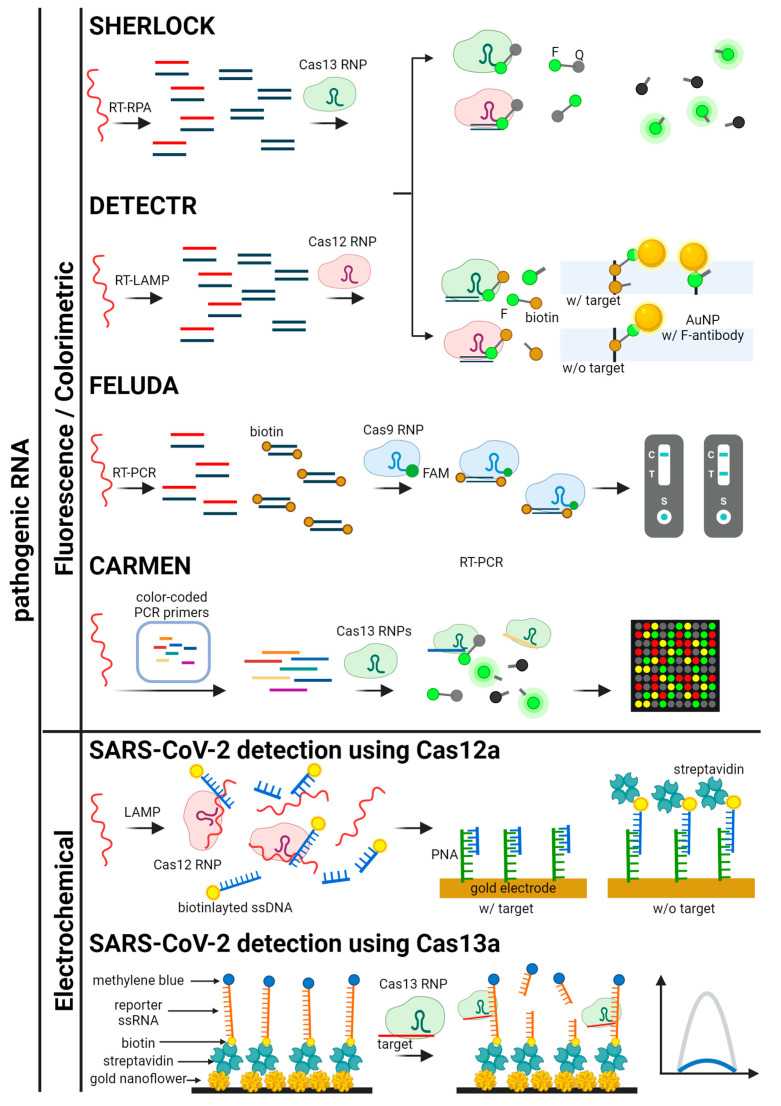
Schematics for CRISPR-based detection systems for pathogenic RNA. The detection systems are categorized by signal readout methods. SHERLOCK, DETECTR, FELUDA, CARMEN, and SARS-CoV-2 detection platforms using Cas12a and Cas13a are illustrated [41,43,46,49,61,77,78].

**Figure 6:**
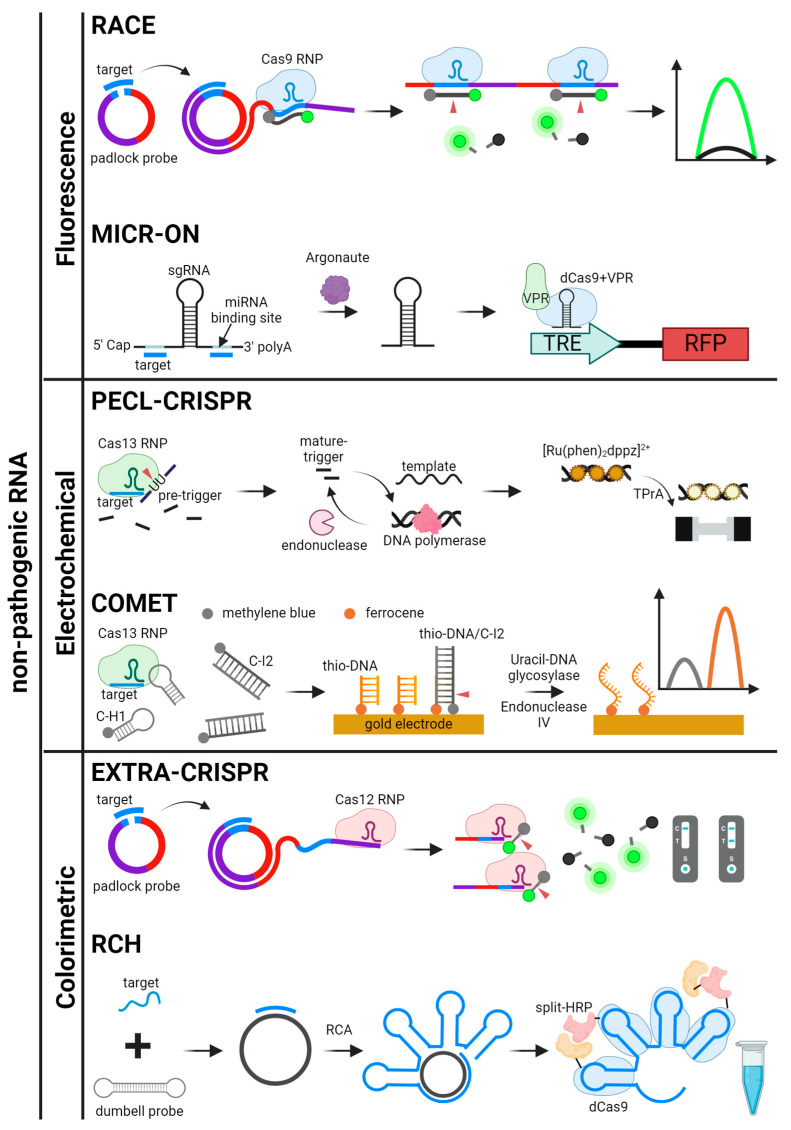
Schematics for CRISPR-based detection systems for non-pathogenic RNA. The detection systems are categorized by signal readout methods. RACE, MICR-ON, PECL-CRISPR, COMET, EXTRA-CRISPR, and RCH are illustrated [50,51,52,53,56,87].

The effectiveness of CRISPR-based nucleic acid detection is closely linked to the signal readout methods, which convert molecular recognition events into measurable signals. This section provides an overview of the primary readout systems used for both pathogenic and non-pathogenic nucleic acids. For pathogenic targets, fluorescence, electrochemical, and colorimetric systems are examined, each offering distinct benefits in terms of sensitivity, simplicity, and adaptability to various diagnostic environments. For non-pathogenic nucleic acids, optical imaging and sensor-based systems are explored, emphasizing their utility in research and precision diagnostics. These readout methods are crucial for ensuring the accuracy and applicability of CRISPR-based detection technologies, underlining their growing importance in molecular diagnostics. Various signal production, readout methods, and detection mechanisms are illustrated in Figure 2 and Figure 3, with graphical descriptions of CRISPR-based nucleic acid detection methods by target types and signal readout systems shown in Figure 4, Figure 5 and Figure 6.

### 4.1. Signal Readout System for Pathogenic Nucleic Acid

The detection of pathogenic nucleic acids via CRISPR-based systems is significantly enhanced by specific signal readout methods. This section introduces three core techniques: fluorescence, electrochemical, and colorimetric readouts. Fluorescence systems utilize the sensitivity of fluorescent signals to identify pathogenic DNA or RNA, making them highly effective in laboratory diagnostics. Electrochemical systems convert molecular interactions into electrical signals, providing rapid, quantitative detection suitable for portable, POC devices. Colorimetric systems offer a simple, visual method for detecting nucleic acids, ideal for quick, on-site diagnostics. The following subsections will detail the applications, benefits, and limitations of each method in the context of pathogenic nucleic acid detection.

#### 4.1.1. Fluorescence Signal Readout Systems

Fluorescence readout systems are widely used in CRISPR-based detection due to their high sensitivity and quantitative capabilities [18,24,29,37,46,61,62,63,64,65,66]. These systems utilize fluorescent reporters that emit light upon activation by CRISPR/Cas-induced cleavage. Foundational pathogen detection platforms that have been paved the way for subsequent technologies include Cas12a-based DETECTR assay and Cas13a-based SHERLOCK assay. Both platforms employ the trans-cleavage activities of Cas12a and Cas13a, wherein the presence of target DNA or RNA activates these proteins to cleave a single-stranded DNA or RNA fluorescent reporter, resulting in a detectable fluorescence signal. The high sensitivity of fluorescence readouts allows for the detection of low-abundance targets, making them suitable for early diagnosis of diseases like viral infections.

Fluorescence readout systems for pathogen detection can be designed using various fluorescent reporters composed of fluorophores and quenchers, which basically operate via fluorescence resonance energy transfer (FRET) mechanism. Incorporating different fluorophores such as FAM and Cy5, along with their respective quenchers, enables the simultaneous detection of two distinct HPV types, 16 and 18 [67]. A recent study demonstrated that integrating a FRET reporter with a microfluidic device, which enhances molecular binding through a high-density micropillar array, improved HPV detection sensitivity by a factor of ten [68]. Furthermore, recently developed digital droplet CRISPR-based assays enable pathogen detection without amplification, by physically generating picolitre-scale droplets [69,70]. These systems detect DNA and RNA pathogens via trans-cleavage mediated by Cas12a and Cas13a, respectively, producing fluorescence signals within 30–75 min, and their compact design makes them suitable for POC applications.

#### 4.1.2. Electrochemical Signal Readout Systems

Electrochemical readout systems offer a robust and portable alternative for CRISPR-based nucleic acid detection [14,27,53,56,71,72,73,74]. These systems measure changes in electrical properties, such as current or voltage, resulting from CRISPR/Cas-mediated cleavage of nucleic acids. Cas9, Cas12a, and Cas13a are frequently used in these setups, where the cleavage event triggers a change in the conductivity or electrochemical activity of a reporter molecule [27,75,76]. Electrochemical sensors can be integrated into POC diagnostic devices, providing rapid and accurate results with minimal sample preparation.

One notable example is the E-CRISPR system, which utilizes Cas12a for the detection of viral nucleic acids, such as HPV-16 and parvovirus B19 (PB-19), with picomolar sensitivity [27]. Single-stranded DNAs labeled with methylene blue are immobilized via thiol moieties on a gold electrode. Upon cleavage of these DNA reporters by activated Cas12a in response to target DNA, an electrochemical signal is generated. Similar strategies have been applied for the detection of SARS-CoV-2, demonstrating significant potential for POC applications [77,78]. This approach provides a portable and accurate solution for POC diagnostics, highlighting the potential of electrochemical readouts in amplification-free CRISPR-based nucleic acid detection. Additionally, the integration of CRISPR-based systems with microfluidic devices further enhances portability, achieving detection limits of 10~10^2^ copies/μL within 75 min, making these systems applicable from clinical laboratories to field diagnostics [79,80].

#### 4.1.3. Colorimetric Readout Systems

Colorimetric readout systems provide a simple and cost-effective method for CRISPR-based nucleic acid detection, making them ideal for use in resource-limited settings. These systems typically involve a visible color change that occurs upon CRISPR/Cas-mediated cleavage of a chromogenic substrate [23,26,28,30,40,42,43,44,45,81,82]. Cas12a and Cas13a are often used in colorimetric assays, where their trans-cleavage activity on a chromogenic reporter generates a color change that can be monitored with the naked eye or quantified using a spectrophotometer.

One of the most widely used colorimetric readout methods is lateral flow assays (LFA) employing gold nanoparticles (AuNPs). In techniques utilizing the trans-cleavage activity of Cas12 or Cas13, AuNPs coated with anti-fluorescein antibodies and dual-labeled single-stranded reporters with fluorescein and biotin at the both ends are used [26,41,63,81]. The control line, coated with streptavidin, captures unprocessed reporters-AuNP complexes, while cleaved fragments with fluorescein-AuNP appear on the test line. By combining isothermal amplification, these methods enable the detection of attomolar concentrations within one hour, demonstrating promising potential for on-site applications. In contrast to Cas12 and Cas13, Cas9 lacks trans-cleavage activity but can still be employed in LFA using AuNPs conjugated with DNA sequences complementary to probes on the control line and the aptamer sequence of gRNA [23]. To facilitate interaction between the target DNA and streptavidin-coated lines, the target sequences are amplified with biotin-labeled primers prior to LFA application. When target DNA is present, the biotinylated targets recognized by Cas9 RNP are tethered to the test line, generating a signal through AuNPs bound to the aptamer structure of gRNA. This approach achieves a sub-femtomolar detection limit with 100% accuracy compared to RT-PCR.

In summary, the versatility of CRISPR-based nucleic acid detection is significantly enhanced by the diverse signal readout methods available. Fluorescence, electrochemical, and colorimetric systems each offer unique benefits, catering to different diagnostic needs and settings. These readout techniques not only improve the sensitivity and specificity of CRISPR diagnostics but also expand their applicability, making advanced molecular diagnostics accessible to a broader range of users.

### 4.2. Signal Readout System for Non-Pathogenic Nucleic Acid

CRISPR-based detection systems have shown remarkable potential not only for identifying pathogenic nucleic acids but also for detecting non-pathogenic nucleic acids, which are crucial for applications in research, diagnostics, and personalized medicine. These systems also rely on signal readout methods to convert molecular recognition events into measurable signals. Among the various optical and electrochemical biosensors employed, merging the capabilities of biosensors with imaging techniques offers a range of benefits, including high sensitivity and specificity.

The integration of optical imaging techniques, such as fluorescence microscopy and bioluminescence imaging, with biosensor platforms enables the visualization of nucleic acids detected by CRISPR-based methods [35,54,83,84,85,86]. These imaging techniques, often enhanced by biosensors, utilize fluorescent or luminescent markers attached to nucleic acids or CRISPR components to provide high-resolution, real-time visualization of molecular interactions. This combination enables the detection of specific biomarkers, such as miRNAs, that are associated with various health conditions.

Detection of regulatory nucleic acids can be categorized into in vivo and in vitro approaches. In vivo detection typically utilizes florescence imaging techniques by introducing biosensors conjugated with fluorescent proteins. MICR-ON measures miRNA levels in vivo using biosensors that operate with red fluorescent protein (RFP) and dCas9-VPR complex [87]. In this method, sgRNAs, made inactive due to the presence of a 5′ Cap and 3′ poly A, contain miRNA-complementary sequences that are processed by Argonaute-mediated cleavage at the miRNA-binding site, allowing for activation. The activated sgRNAs then form a complex with dCas9-VPR, initiating transcription and resulting in RFP expression. This approach enables monitoring of miRNA levels with high specificity, and it can even facilitate simultaneous detection of two distinct miRNAs using two sgRNAs that separately activate RFP and green fluorescent protein (GFP).

In vitro detection can employ various signal readout methods, including fluorescence, electrochemical, and colorimetric readouts. Typically, target nucleic acids are amplified, and the Cas-gRNA complex binds to multiple targets, generating measurable signals in a manner similar to pathogenic detection. A notable approach that avoids conventional amplification methods utilizes a quenching effect between fluorophores and AuNPs positioned less than 2 nm apart [88]. Two differently sized AuNPs are connected by partially single-stranded DNA labeled with a fluorophore, which is quenched by the larger AuNP. Upon cleavage of the single-stranded region by activated Cas12a in the presence of target DNA, the released fluorophore-labeled DNA-conjugated small AuNP generates fluorescence, further enhanced by the small AuNP. This method achieves sub-femtomolar detection limit without amplification, which is three orders of magnitude more sensitive than conventional reporter systems.

## 5. Comparative Performance of CRISPR-Based Detection with Traditional Approaches for Viral Detection

A comparison of CRISPR-based nucleic acid detection with traditional diagnostic approaches such as real-time quantitative PCR (qPCR), isothermal amplification, and enzyme-linked immunosorbent assays (ELISA) provides valuable insights into the advantages and limitations of these technologies. Although CRISPR-based techniques have emerged as a powerful tool for viral detection, traditional methods continue to serve as the gold standard for clinical diagnostics due to their high sensitivity and broad applicability. This section evaluates the performance of CRISPR-based methods relative to conventional techniques, particularly in terms of sensitivity, time-to-result, and suitability for POC applications.

Traditional methods like qPCR are highly regarded for their sensitivity and specificity in detecting viruses such as SARS-CoV-2. qPCR remains one of the most reliable diagnostic tools available, but it comes with limitations. The need for skilled technicians, specialized equipment such as thermocyclers, and time-consuming workflows restricts its applicability in resource-limited POC settings. Additionally, qPCR often requires 3–4 h to provide results, which can be a bottleneck in rapid diagnostic scenarios.

Isothermal amplification techniques such as LAMP provide a faster alternative by allowing nucleic acid amplification by eliminating the need for thermal cycling. For example, RT-LAMP can detect SARS-CoV-2 within 30–60 min, making it more practical for rapid diagnostics. However, isothermal amplification methods present tends to have lower sensitivity compared to qPCR and are more prone to false positives due to nonspecific amplification.

Antigen-based methods, such as ELISA, detect viral proteins or host antibodies rather than nucleic acids, are often used in rapid antigen tests, and are widely available through LFA platforms. Despite its rapid results, ELISA generally exhibits lower sensitivity, particularly during the early stages of infection when antigen levels are insufficient for detection.

CRIPSR-based methods, such as SHERLOCK and DETECTR, combine the advantages of isothermal amplification with the precision of CRISPR-mediated target recognition. These techniques offer higher specificity than ELISA, with the ability to discern viral mutations and flexibility in adapting to new viruses by redesigning gRNA. Moreover, CRISPR-based assays can deliver results within an hour, and their integration into POC platforms is more straightforward than qPCR, making them a promising option for rapid diagnostics.

While CRISPR-based techniques hold great promise, especially in terms of speed and adaptability, qPCR remains the most sensitive and reliable method for clinical diagnostics. Continued optimization of CRISPR-based approaches will be required to unlock their potential as mainstream diagnostic tool for viral detection.

## 6. Challenges in CRISPR-Based Nucleic Acid Detection

As listed in Table 3, CRISPR-based nucleic acid detection has shown tremendous potential in various applications, but several challenges still need to be addressed to fully harness its capabilities. Key challenges include the amplification of target nucleic acids, achieving multiplexed detection, and ensuring accurate quantitative detection. These issues affect the sensitivity, specificity, and practicality of CRISPR-based diagnostics, necessitating continuous innovation and refinement.

### 6.1. Amplification of Target Nucleic Acids

Amplification is a critical step in most CRISPR-based nucleic acid detection methods to enhance sensitivity. Traditional methods like PCR and LAMP are commonly employed to increase the quantity of target nucleic acids before detection. However, these techniques can introduce complexity, extend the detection time, and require sophisticated equipment, which limits their applicability in POC settings. For example, PCR involves thermal cycling, which necessitates expensive and bulky thermal cyclers, making the process less suitable for rapid, field-deployable diagnostics.

To address these limitations, researchers are developing amplification-free CRISPR detection methods [95,96]. These methods leverage the high specificity of CRISPR/Cas systems to detect target nucleic acids directly without prior amplification. One example is a Cas13a-based detection platform that integrates a catalytic hairpin DNA circuit to generate signaling molecules, which subsequently bind to DNA on an electrode [53]. This approach has enabled the detection of microRNAs via electrochemical signals, achieving a detection limit of 50 aM within 36 min, without conventional amplification. Another amplification-free CRISPR-based detection method involves targeting non-coding RNAs using LFA that senses Cas13-triggered trans-cleavage products through ELISA with catalytic nanoparticles [97].

The development of these amplification-free techniques non only improves detection sensitivity but also reduces the risk of non-specific amplicons and carryover contamination, making CRISPR-based diagnostics more suitable for on-site detection.

### 6.2. Off-Target Effects

CRISPR-Cas systems inherently exhibit off-target effects, where unintended cleavage occurs at nucleic acids that share sequence similarity with the intended target. Although CRISPR-Cas systems are highly specific, mismatches between the gRNA and non-target sequences can still lead to undesired detection events, potentially compromising the accuracy and reliability of CRISPR-based detection. These off-target effects may result in false-positive results, with the risk particularly increasing in multiplexed detection, where the potential overlap in sequence homology among various targets becomes more pronounced.

To reduce off-target effects in CRISPR-based nucleic acid detection, several strategies can be employed. These include optimizing gRNA design to enhance target specificity, utilizing engineered Cas proteins with higher fidelity, and employing computational tools to predict and avoid off-target sites. For instance, the DNA extension to crRNA of Cas12a has been shown to improve specificity by up to 8.8-fold across various off-targets [98]. Additionally, incorporating universal bases into the gRNAs of Cas9 enables the simultaneous targeting of polymorphic sequences, and applying this strategy to Cas12a-based DETECTR platform has allowed for the identification of HIV protease variants [99]. Ultimately, further advancements in gRNA and Cas protein engineering are expected to improve the robustness of CRISPR-based nucleic acid detection systems, making them more reliable for clinical diagnostics and pathogen surveillance.

### 6.3. Multiplexed Detection

Multiplexed detection enables the simultaneous identification of multiple target sequences in a single reaction, which is crucial for comprehensive diagnostics [100,101]. However, achieving multiplexing in CRISPR-based systems presents significant challenges. The primary issue is the potential for cross-reactivity and signal interference when detecting multiple targets simultaneously. For instance, in systems using Cas13 for RNA detection, the collateral cleavage activity can cause non-specific degradation of nearby RNA molecules, leading to false positives and compromised specificity.

Several strategies are being explored to overcome these challenges [47,61,102]. One approach involves using orthogonal Cas proteins that recognize different PAM sequences or spacer regions, thus minimizing cross-reactivity. Another method is to physically separate the detection reactions within microfluidic devices or using spatially distinct signal transduction elements, such as different fluorophores or electrochemical sensors. Advanced platforms like SHERLOCKv2 and CARMEN-Cas13a have demonstrated the potential for high-throughput and multiplexed pathogen detection by integrating multiple CRISPR systems and readout techniques. Nevertheless, the simultaneous detection of multiple targets from a single sample remains an unsolved challenge.

Future advancements in multiplexed detection may be inspired by improvements in digital PCR (dPCR). Recent breakthroughs in multiplex digital PCR have demonstrated the potential of artificial intelligence (AI). These advances include deep-learning approaches for distinguishing similar colors from a single fluorescent channel, in silico methods for optimizing multiplex assays, and all-in-one workflow combining oscillation-driven droplet generation with AI-based analysis [103,104,105]. Since dPCR shares similar working principles with multiplexed detection using CRISPR, the integration of AI-assisted models into CRISPR-based nucleic acid detection could enable the simultaneous identification of multiple targets from a single sample [106]. These innovations would pave the way for more robust and scalable multiplexed CRISPR diagnostics.

### 6.4. Quantitative Detection

Quantitative detection of nucleic acids is essential for applications that require precise measurement of target concentrations, such as monitoring viral loads, detecting SNPs, and measuring gene expression levels. However, traditional CRISPR-based assays like SHERLOCK and DETECTR are primarily qualitative, indicating the presence or absence of target nucleic acids without providing quantitative data.

To achieve quantitative detection, CRISPR-based methods are being combined with techniques such as real-time PCR, digital PCR, and NGS. These combinations allow for the quantification of nucleic acids by correlating the CRISPR-mediated cleavage events with the amplification cycles or sequencing reads. Additionally, digital CRISPR methods, such as droplet microfluidics, partition the sample into numerous small reactions, enabling absolute quantification by counting the positive reactions. This approach enhances sensitivity and provides precise quantitative measurements of low-abundance targets. Continued development of these integrated platforms will be crucial for expanding the quantitative capabilities of CRISPR-based diagnostics.

In summary, while CRISPR-based nucleic acid detection offers promising advancements, addressing the challenges of target amplification, multiplexed detection, and quantitative analysis is vital for its broader application. Innovative solutions and technological integrations are essential to overcome these challenges and fully unlock the potential of CRISPR diagnostics in various fields.

## 7. Conclusions and Outlook

The emergence of CRISPR/Cas systems has revolutionized nucleic acid detection, offering high specificity and sensitivity. Despite the remarkable progress made, several challenges remain that must be addressed to fully realize the potential of CRISPR-based diagnostics. Key areas for improvement include the need for amplification-free detection methods, enhanced multiplexing capabilities, and accurate quantitative measurements.

For the detection of pathogenic nucleic acids, future developments in CRISPR technology will likely focus on optimizing systems for better performance in diverse and practical settings. This includes the integration of portable microfluidic-based POC devices that carry all reagents onboard. However, the multi-step procedures, such as clinical sample pretreatment and prior amplification, present challenges for practical use. Although these approaches improve sensitivity, the complexity of the workflow, the risk of cross-contamination, and the need for technical expertise prevent their translation from laboratory to on-site applications. One-pot reactions using engineered Cas proteins have recently demonstrated improvements in efficiency and sensitivity [107,108]. Moving forward, further enhancements in sensitivity, achieved by engineering Cas proteins or optimizing gRNAs, will be essential to reaching detection limits comparable to gold-standard techniques such as qPCR. Such advancements will make CRISPR-based diagnostics more accessible and easier to use outside of laboratory environments.

In the context of non-pathogenic nucleic acid detection, there is growing interest in detecting intracellular nucleic acids such as non-coding RNAs, mitochondrial DNA, and cytosolic DNA, for disease diagnostics. Imaging these nucleic acids in live cells is mostly performed using catalytically inactive Cas9 fused with transcriptional effectors. A major challenge in this area is the low abundance of these intracellular nucleic acids, which necessitates the use of signal amplification strategies. However, the application of trans-cleavage activity, an intrinsic amplification feature of Cas12, Cas13, and Cas14, is limited by the diffusion-mediated nature of interactions within cells. Future work could focus on optimizing reporter concentrations and employing physical confinement strategies to enhance the applicability of trans-cleavage activity for intracellular nucleic acid detection.

In addition, minimizing off-target effects, improving the fidelity of CRISPR/Cas systems, and expanding PAM recognition to target a broader range of sequences remain important goals. Developing high-fidelity Cas proteins and optimizing guide RNA sequences are crucial steps towards achieving these objectives. Moreover, efforts to streamline the detection process could further simplify the workflow and reduce the risk of contamination.

In conclusion, while CRISPR/Cas-based nucleic acid detection systems have already made significant strides, continuous innovation and optimization are necessary to overcome existing challenges. By addressing these issues, CRISPR technology would become a cornerstone of molecular diagnostics, offering rapid, sensitive, and specific detection solutions that can be widely applied across various fields, from medical diagnostics to environmental monitoring.

## Figures and Tables

**Table 1:** Overview of properties of Cas proteins commonly used in CRISPR-based nucleic acid detection.

Cas Protein	Cas9	Cas12	Cas13	Cas14
Cas length (a.a.)	1000–1700	1200–1300	~1200	400–700
Nuclease domain	HNH and RuvC	RuvC	2xHEPN	RuvC
Spacer length	18–24 nt	18–25 nt	22–30 nt	20–40 nt
PAM(PFS)	NGG	TTTV	non-G	None
Target type	dsDNA	dsDNA/ssDNA	ssRNA	dsDNA/ssDNA
Trans cleavage	None	ssDNA	ssRNA	ssDNA

**Table 2:** Amplification techniques used in CRISPR-based nucleic acid detection.

Methods	PCR	RPA	RCA	LAMP	SDA	NASBA
Temperature (°C)	95, 50–65, 72	37–42	20–37	60–65	37–60	41
Time (min)	120–180	10–60	90–120	20–60	60–120	30–120
Number of primers	2	2	2	4 or 6	2 or 4	2
Other proteins besides polymerase	-	Recombinase, single-strand binding protein	Ligase	-	Restriction enzyme	Reverse transcriptase, RNase H

“-“: not applicable; PCR: polymerase chain reaction; RPA: recombinase polymerase amplification; RCA: rolling circle amplification; LAMP: loop-mediated isothermal amplification; SDA: strand displacement amplification; NASBA: nucleic acid sequence-based amplification.

**Table 3:** Overview of CRISPR-based nucleic acid detection platforms for diagnostics.

Target	Cas Protein	Platform	Amplification	Readout	Application	Ref.
DNA	Cas9	ctPCR	PCR	PCR	HPV	[19]
		CAS-EXPAR	EXPAR	Fluorescence	DNA methylation, *Listeria monocytogenes*	[22]
		CARP	-	qPCR	HPV	[20]
		CASLFA	PCR/RPA	Colorimetric	ASFV, *Listeria monocytogenes*	[23]
		CRISPR-Chip	-	Electrochemical	SNP	[71]
		CRISDA	SDA	Fluorescence	SNP	[90]
		FLASH	PCR	NGS	Antimicrobial resistance (AMR)	[91]
		GPHOXE	-	Impedance	circulating tumor DNA	[34]
		CasPLA		Fluorescence	mitochondrial DNA	[35]
					
	Cas12	DETECTR	RPA	Fluorescence	HPV	[24]
		HOLMES	PCR	Fluorescence	Pseudorabies virus (PRV), SNP	[37]
		CIA	LAMP	Colorimetric	HPV, *Pseudomonas aeruginosa*	[26]
		E-CRISPR	-	Electrochemical	HPV, Parvovirus B19 (B19V)	[27]
		CLIPON	RPA	Colorimetric	HPV	[28]
		CDetection	RPA	Fluorescence	HPV	[29]
		CORDS	RAA	Colorimetric	ASFV	[30]
		PEC-CRISPR/Cas12a	-	Electrochemical	HIV-DNA	[72]
		Cas12a-VDet	RPA	Fluorescence	Mycoplasma	[92]
					
	Cas13	CCB-Detection	PCR	Fluorescence	*Staphylococcus aureus*	[62]
					
	Cas14	Cas14-DETECTR	PCR	Fluorescence	SNP	[18]
						
						
RNA	Cas9	FELUDA	RT-RPA	Colorimetric	SARS-CoV-2	[43]
		NASBACC	NASBA	Colorimetric	Zika	[40]
		RCasFISH	-	Fluorescence	messenger RNA	[54]
		RCH	RCA	Colorimetric	miRNA	[50]
		RACE	RCA	Fluorescence	miRNA	[51]
					
	Cas12	DETECTR	RT-LAMP	Fluorescence	SARS-CoV-2	[41]
		CASdetec	RT-RAA	Colorimetric	SARS-CoV-2	[42]
		VaNGuard	RT-LAMP	Colorimetric	SARS-CoV-2	[44]
		AIOD	RT-RPA	Colorimetric	SARS-CoV-2, HIV	[45]
		EXTRA-CRISPR	RCA	Colorimetric	miRNA	[52]
					
	Cas13	SHERLOCK	RT-RPA	Fluorescence	Zika, Dengue virus	[46,49]
		CARMEN	PCR/RPA	Fluorescence	RNA viruses	[61]
		CARVER	RT-PCR	Fluorescence	Influenza, Lymphocytic choriomeningitis virus	[93]
		COMET	-	Electrochemical	RNA, miRNA	[53]
		SHINE	RT-RPA	Fluorescence, Colorimetric	SARS-CoV-2	[82]
		CREST	PCR	Fluorescence	SARS-CoV-2	[63]
		PECL-CRISPR	EXPAR	Electrochemical	miRNA	[56]
						
	Cas14	Cas14SDA	SDA	Fluorescence	miRNA	[94]

“-“: not applicable; RAA: recombinase aided amplification; RT: reverse transcription; EXPAR: Exponential amplification reaction; NGS: next generation sequencing; HRP: Horseradish peroxidase; miRNA: microRNA; SARS-CoV: severe acute respiratory syndrome coronavirus; HIV: human immunodeficiency virus; HPV: human papilloma virus; AFSV: African swine fever virus; SNP: single nucleotide polymorphism.

## Data Availability

No new data were generated in this study.
